# Editorial: Cerebellar computations across the lifespan

**DOI:** 10.3389/fncom.2026.1872573

**Published:** 2026-05-28

**Authors:** Marlies Oostland, Jessica L. Verpeut

**Affiliations:** 1 Swammerdam Institute for Life Sciences, University of Amsterdam, Amsterdam, Netherlands; 2 Department of Psychology, Arizona State University, Tempe, AZ, United States

**Keywords:** aging, autism, cerebellum, circuits, cognition, computation, social behavior, spinocerebellar ataxia

## Introduction

Cerebellar guidance of brain-wide circuits can influence cognitive and social functions, which are evolving across the lifespan: during early life there is development of cognitive and social skills, while neurodegenerative disorders in aging are often hallmarked by a loss of cognitive and social functions. Due to the vast bidirectional connections between the cerebellum and rest of the brain, cerebellar dysfunction can have brain-wide, long-lasting, and diverse consequences, including but not limited to neurodevelopmental disorders and aging dementias. There is a clear need to also consider cerebellar contributions beyond motor domains, a previous reductionist view of cerebellar function. The collection of papers in this Research Topic aims to contribute an evolving insight into cerebellar computations across the lifespan, from synapse to clinic.

## Structure and plasticity of cerebellar microcircuits

First, the paper by Han et al. addresses cerebellar circuits at the synaptic level, by demonstrating that mossy fiber to granule cell connections do not occur randomly, but have a structured pattern to allow molecularly distinct inputs to each granule cell. Using a volumetric correlated light and electron microscopy (vCLEM) dataset of the adult mouse cerebellum, they identified VGluT1-positive and VGluT1-negative mossy fiber terminals and reconstructed connections between these mossy fibers and granule cells. Interestingly, individual granule cells preferentially connect to specific classes of mossy fibers, suggesting that these structures have a planned developmental pattern. These results refine long-standing theories of cerebellar development, suggesting structured input selection and probabilistic output organization to support efficient computational processing.

Next, integrating multiscale data, Santoro et al. provides a comprehensive review of computational models of Purkinje cells. From single-cell representations to complex synaptic and extrasynaptic input models, the reviewed work demonstrates how computational approaches can reproduce physiological features of Purkinje cell activity. Uniquely this review incorporates both somatic and dendritic models, capturing the richness of the cerebellar microcircuit. There is a need in neuroscience for integrative, mechanistic models to link cellular properties and circuit synaptic to model behavior and disease. These computational frameworks will be essential for diagnostics and treatment of cerebellar-related disruptions.

## Cognitive, social, and affective implications of cerebellar dysfunction across the lifespan

Understanding microcircuit development of the cerebellum is foundational to the work by Cundari et al. which examined cerebellar disruptions beyond motor control. In this study, individuals with Spinocerebellar Ataxia Type 34 (SCA34) were analyzed using high-resolution 7T MRI alongside neuropsychological testing. SCA34 was found to be characterized by both motor and cognitive dysfunction, aligning with known features of cognitive-affective syndrome (CCAS). It is important to note that clinical usage of CCAS testing is waning, but this critical study cautions moving away from these diagnostic criteria. The authors further identify region-specific cerebellar atrophy, adding further data to the growing body of cerebellar literature linking structural degeneration and functional impairments. The majority of clinical data thus far has failed to focus on female participants and here the authors show critical sex-specific cerebellar contributions to anxiety and sleep. Together these findings demonstrate cerebellar circuit-level dysfunction and complex changes to behavior is cerebellar region-dependent and sex-specific across the lifespan.

Finally, in our own opinion article, we highlight the importance of early-life cerebellar development for shaping brain function and behavior (Verpeut and Oostland). Disruptions during critical developmental windows, which are not all yet clearly defined in the cerebellum, have long-lasting effects on cerebellar circuitry and distal cortical brain structures. These disruptions, whether genetic or environmental, shape the adult brain in diverse ways. How these diverse perturbations impact the cerebellum across the lifespan and into aging is unclear. The question remains how the cerebellum is able to exert this influence on brain-wide networks and give rise to flexible yet complex computations.

## Conclusion

Together, this Research Topic emphasizes that cerebellar computations are not random and allow for a level of plasticity that emerges through structured connectivity during development. From the detailed explanations of selective granule cell wiring to system-wide genetic disruptions, to Purkinje cell circuit dynamics, these studies are a unique Research Topic on a lifespan perspective on cerebellar function. Future work in high-resolution connectomics, sex-disaggregated data, longitudinal cerebellar imaging or recording, and computational models will allow for a larger understanding of how cerebellar circuits influence behavior and how perturbations to these networks lead to disorders.

